# Effects of a computerized psychological inoculation intervention on condom use tendencies in sub Saharan and Caucasian students: two feasibility trials

**DOI:** 10.1080/21642850.2019.1614928

**Published:** 2019-05-15

**Authors:** Einav Levy, Yori Gidron, Reginald Deschepper, Benjamin O Olley, Koen Ponnet

**Affiliations:** a Faculty of Medicine and Pharmacy, Free University of Brussels, Brussels, Belgium; b The Israeli School of Humanitarian Action, Tel Aviv, Israel; c SCALab, Lille3 University, Lille, France; d Department of Psychology, University of Ibadan, Ibadan, Nigeria; e Department of Communication Sciences, imec-mict-Ghent University, Ghent, Belgium

**Keywords:** Condom use, barriers, psychological inoculation, HIV, sub-Sahara Africa, automatized system

## Abstract

**Objective:** An effective method for preventing the Human Immunodeficiency Virus (HIV) is condom use. Yet, research shows limited effects of education on increasing condom use. This research examined the effects of psychological inoculation (PI) versus education on condom use -barriers and –tendencies, using a fully automatized online system.

**Design:** Two randomized controlled trials. In Study 1, 59 Sub-Saharan students were included while Study 2 20 European students were included. In both studies, participants were randomly assigned to PI or control conditions. In Study 2, we additionally matched pairs on gender and condom barriers. In the PI, participants received challenging sentences they had to refute.

**Main outcome measures:** An indirect condom use test (I-CUTE) and a condom use barriers questionnaire, assessed at baseline and a month later.

**Results:** In Study 1, a significant increase in I-CUTE scores and no change in barriers was found in the PI condition. Controls did not change on either outcome. In Study 2, two sub-scales of condom barriers (concerning partner and satisfaction) were significantly decreased in the PI group, while in controls, barriers significantly increased over time. In both groups, I-CUTE scores tended to increase.

**Conclusions:** These results replicate previous studies and extend them to a fully automatized system without counselors.

## Introduction

Human immunodeficiency virus (HIV) causes Acquired Immune Deficiency Syndrome (AIDS), which is one of the most fatal diseases known to humankind and is one of the major causes of years of life lost (GBD, 2016, Causes of Death collaborators, 2017). The estimated number of people living with HIV is 37.7 million worldwide and about 25.5 million are estimated to reside in Sub Sahara Africa alone. In 2015, it was estimated that 2.1 million people were newly infected by HIV. From these newly infected people, 1.4 million were in Africa alone (http://www.unaids.org, 2016; http://www.who.int, 2016).

Studies show that the prevalence of HIV is declining, with exceptions in East Europe and Central Asia among people who inject drugs, and in young women in Sub-Saharan Africa and young men who have sex with men (Beyrer & Abdool Karim, 2013). In parallel to these trends, the introduction of Anti-Retroviral Therapy (ART) has reduced the mortality from HIV in some African countries (Chihana et al., 2012). Additional effective pharmacological approaches for reducing HIV transmission include Pre Exposure Prophylaxis (PrEP) (Cohen et al., 2015; Haberer et al., 2014; Murnane et al., 2015; Naswa & Marfatia, 2011; Thomson et al., 2016). While clear advancements have taken place in the treatment of HIV, these should be complimented by efforts to prevent it, to begin with.

A major path to the prevention of HIV is the practice of protected sex, which reflects behavioral approaches. Indeed, studies have shown that persistent condom use reduces the risk of contracting HIV. Specifically, in a systematic review of 14 studies, consistent use of condoms was associated with an 80% reduction of HIV risk (Weller & Davis-Beaty, 2002). One of the main methods previously used for increasing condom use is health education. Yet, studies have shown that education alone has little impact or statistically non-significant effects on the consistent use of condoms (Gallant & Matika-Tyndale, 2004). The latter research reviewed 11 intervention studies performed on African youth. The most consistent findings were improvements in attitudes and knowledge. However, only two studies targeted actual condom use, in which only in one was there improvement in this outcome. Two main reasons for the limited effects of health education may be the fact that education does not alter cognitive barriers and misbelieves which underlay condom non-use, and it does not teach people to resist social pressures against condom use (Gallant & Matika-Tyndale, 2004).

There are several models of behavior change which include and can guide researchers to focus on such cognitive and social factors. Two main social cognitive models (SCM), which have also been tested in relation to condom use, are the health belief model (HBM; Rosenstock, 1974) and the theory of planned behavior (TPB; Ajzen, 1991). Briefly, the HBM states that people’s perceptions about the susceptibility and severity of a disease, together with perceived barriers against adopting preventative behaviors and their perceptions of benefiting from them, predict the eventual adoption of health behaviors. Indeed, there is evidence that this model explains variance in condom use (Asare, Sharma, Bernard, Rojas-Guyler, & Wang, 2013). Thus, this model relates to the condom-use barriers mentioned above.

The TPB posits that people’s attitudes about health behaviors, their perceived social norms (e.g. what society or meaningful others think about these behaviors) and their own behavioral control over adopting a behavior – all predict people’s intention and eventual adoption of a health behavior. Concerning condom use, Eggers et al. (2016) for example found in three Sub Saharan African (SSA) regions that various elements of the TPB predict intentions and to a lesser extent actual condom use. Thus, this model relates to the social pressures mentioned above.

Together, social-cognitive models represent human control and self-regulation mechanisms, which involve expectations about the potential outcomes resulting from performing a certain behavior and the subsequent adoption of behaviors (Luszczynska & Schwarzer, 2005).

Studies have identified multiple cognitive barriers people may have against the use of condom. These barriers are including price, reduced pleasure and perceived fragility of the condom (Adih & Alexander, 1999), anticipated health problems resulting from condom use (Gallagher et al., 2014; Sunmola, 2005) and hindered sexual interest (Sunmola, 2005).

Studies on specific social pressures which prevent people from using condoms showed that these barriers include subjective norms about significant others’ opinions concerning condoms (Brüll, Ruiter, Wiers, & Kok, 2016; Janepanish, Dancy, & Park, 2011). Indeed, one meta-analysis found that the intention to use condoms was related to such subjective norms (Albarracin, Johnson, Fishbein, & Muellerleile, 2001). Social environmental pressures include for example perceived marital infidelity, trust in partner, cultural and religious attitudes as reported by African Caribbean women (Baidoobonso, Bauer, Speechley, & Lawson, 2013), and lack of privacy in stores and social stigma when purchasing condoms, as reported in Mumbai, India (Roth, Satya, & Bunch, 2001). Futhermore, the negative effects of social stigma and attitudes towards people living with HIV, resulted in lower condom use among women in Nigeria (Lammers, van Wijnbergen, & Willebrands, 2013).

These social cognitive factors can be targets of interventions for increasing condom use beyond the provision of knowledge and health education alone. A few intervention studies also targeted such cognitive and social factors (Heeren, Jemmott, Ngwane, Mandeya, & Tyler, 2014; Mathews et al., 2012; Pronyk et al., 2006, 2008; Visser, 2007). A recent meta-analysis found that interventions using these psychoeducational methods reduced STD’s (Wariki et al., 2012). One important method, which targets such factors for increasing condom use, is motivational interviewing. This method helps people identify and reduce their own barriers, and was found to be effective in reducing HIV risk behavior (Nugroho, Erasmus, Zomer, Wu, & Richardus, 2017). However, among the limitations of these interventions are their duration, limited ability to administer them en-masse and the difficulty to adapt them to different cultures.

An alternative method which addresses all these limitations and which targets cognitive barriers and social pressures is psychological inoculation (PI). This method may integrate elements from both of the theories described above, as barriers appears in the HBM and social norms in the TPB. The PI method includes exposing people to challenging sentences (i.e. ‘the vaccine’), which reflect the internal cognitive barriers and external social pressures about condom use, in an exaggerated manner. People are then guided to systematically reject the sentences (i.e. ‘the antibody response’; Duryea, Ransom, & English, 1990). PI was found to prevent smoking, prevent joining a driver who consumed alcohol and to reduce driving hostility and traffic accidents in simulated driving (Duryea et al., 1990; Gidron, Zack Slor, Toderas, Herz, & Friedman, 2015). Of greatest relevance to the present study, a pilot study found that PI significantly reduced condom-use barriers, increased condom negotiation self-efficacy and tended to increase self-reported condom-use, in Nigerian women with HIV, while an education control condition did not have such effects (Olley, Abbas, & Gidron, 2011). However, that pilot study included a small sample (*N* = 22), a very short follow up (a week) and lacked a valid measure of condom use. In addition, previous studies assessed condoms via self-report methods, which in such contexts, are heavily biased by social desirability. A newly developed semi-projective tool, the Indirect Condom Use Test (I-CUTE), tries to specifically overcome this issue (Levy, Gidron, & Olley, 2017), and was used in the present study.

While a recent meta-analysis found that behavioral interventions were effective in increasing condom use, such interventions required a counselor (Scott-Sheldon, Huedo-Medina, Warren, Johnson, & Carey, 2011). In addition, the PI pilot study of Olley et al. (2011) relied on therapist contact. These reduce the applicability to en-masse interventions for trying to achieve wide spread HIV prevention globally. This may require rethinking on developing a method without a therapist. Thus, a new type of intervention, addressing all these limitations, is needed.

The present study aimed to address many of these past limitations. The purpose of this research was to test the effects of PI on a larger sample, with a longer follow up and with a valid measure of condom use tendencies, which tries to reduce self-report biases. Furthermore, in the present studies, the interventions were given via a fully automatized online system, without any counselor contact. This increased the applicability of our intervention for en-masse administration in the future, should the intervention be effective.

We hypothesized that PI would significantly reduce condom use barriers and increase condom use tendencies, better than a health education control condition. Furthermore, we hypothesized that the degree of refuting the challenging sentences would correlate with the degree of change in barriers and condom use (Study 2).

## Study 1

### Method

#### Participants

The study sample was recruited from a total population of 530 Sub- Saharan students who took part in an educational program in Israel, and to whom the study was proposed. All the students were English literate and were preselected to the educational program according to academic criteria of the program (Grades and academic achievements). The minimal age was 18 years old. The study was approved by the directors of the participants’ educational program in Israel, who reviewed the study and its protocol. All 530 students were informed about the study by email and by their program directors, emphasizing their anonymity and voluntary participation. Initially 208 students entered the website and completed the base-line assessments (Phase one). Of these, 59 had completed *both* base-line and one-month follow-up assessments, which constitutes 24.8% of those initially entering the website. Participants provided their consent to take part in the study electronically, after being informed who are the researchers, the study aims, their right to participate voluntarily and their anonymity. The students were informed that they could withdraw from the study at any time, without any consequences for their studies.

To calculate the sample size, our primary outcome was the I-CUTE scores. We derived the anticipated mean (and SD) of the I-CUTE from Levy et al. (2017) which was conducted on Sub-Saharan students. Assuming 10–15% higher I-CUTE score in the PI group compared to controls after treatment, a statistical significance of *p* < 0.05 and statistical power of 0.80, the total required sample size was 34–78 (www.stat.ubc.ca). Thus, the enrollment of 59 participants recruited for this pilot RCT met these calculations.

#### Measures

The entire study was conducted by an automatized on-line system. The system was programed such that it was not possible to continue to the next item or question without completing the previous one. This prevented problems of missing data.

*Sociodemographic variables*: These included age, gender, having a partner, having sexual relations with the partner and using a contraceptive. The issue of having a partner and sexual relations were assessed in detail by including four response categories: No partner, partner without sexual relations, several partners with sexual relations, and one partner with sexual relations. These categories were mutually exclusive.

*Condom use tendencies*: This was assessed by the indirect condom use test (I-CUTE; Levy et al., 2017). This test was based on the method of the Rosenzweig Picture Frustration Test (RPFT; Rosenzweig, 1978). The validated I-CUTE includes 17 pictures depicting people either in intimate proximity (non-erotic) or with a condom. Under each picture, there was a question asking the participant what one of the characters *in the picture* was thinking or doing, reflecting the projective element of the RPFT, projection from the participant to the person in the picture. Below the question were four responses. Participants had to choose one of the four responses, which reflected their own tendencies to use condoms, from low (1) to high (4). In some pictures, the responses were from high to low, to avoid response sets. After recoding the reversed item responses, the responses were summed up to obtain the total condom use tendency score. Thus, higher scores reflected greater tendency to use condoms. The scenarios were chosen to reflect issues pertinent to condom use, and included attitudes and awareness to health risks, relationships, commitment and trust (Setsuko Hendriksen, Pettifor, Lee, Coates, & Rees, 2007). This semi-projective tool was developed to reduce self-reported biases. Moreover, this tool was developed in accordance with gender-related issues discussing relationship power, gender inequality and decision making, all relevant to condom use in different cultures (Bauermeister, Hickok, Meadowbrooke, Veinot, & Loveluck, 2014; Levy et al., 2017; Olley et al., 2011; Pulerwitz, Amaro, Jong, Gortmaker, & Rudd, 2002). In its development, the I-CUTE had an adequate internal reliability (a Cronbach’s Alpha of 0.74), and its scores correlated significantly with condom use barriers, and did not correlate with social desirability. These findings supported the I-CUTE’s reliability, convergent and discriminate validity, respectively. In the present study, the internal reliability reached a Cronbach’s Alpha of 0.71. The test-retest reliability of the I-CUTE scores within the control group over one month was high (*r* = 0.68, *p* < 0.001).

*Condom use barriers*: This construct was assessed by the condom Use Barriers scale (St. Lawrence et al., 1999). This scale contains 28 items, which relate to health, economic, religious and partners’ barrier domains concerning condoms. In the present study, the internal reliability was high (Cronbach’s alpha = 0.92). Examples of items from the scale are: ‘It is up to the man to provide a condom’ OR ‘I would be afraid to ask my partner to use a condom’. Each item was rated on a 1 to 5 scale, with 1 = ‘Strongly disagree’, and 5 = ‘Strongly agree’. Thus, higher scores reflected more perceived barriers.

### Interventions

The study included two intervention groups, an experimental and a control group. Both groups had received an educational component. This component included background information about prevalence and risk factors of HIV and prevention methods, provided on a screen. The control group additionally received 10 questions on condom use, to control for the interactive element used in the PI group. The 10 True/False questions for controls focused on the knowledge of the participant after receiving general information about Condom use and HIV. An example of a question was: ‘You can put a condom on any time before ejaculation, even at the last seconds’ OR ‘HIV/AIDS is prevalent only among Homosexual populations or sex workers’.

The experimental group had received PI, following Olley et al. (2011). This contained challenging sentences (reflecting common condom use barriers or social pressures), and three refutation sentences, given in advance to the participant. An example of a challenging sentence is: ‘You have never had Sexual Gratification while using a condom’. Each challenging sentence reflected a barrier, which the participant had to reject according to his/her perception. The provided refutation sentences were pre-selected and scaled from no refutation, through partial refutation, to full refutation. The refutation options for this example were: (1) ‘I have never had Sexual gratification while using a condom’ (poor refutation), (2) ‘ I’m not sure I had Sexual Gratification while using a condom’ (partial refutation), and (3) ‘Of course I had sexual gratification while using a condom’ (full refutation). If a participant had chosen the strong refutation, the computer provided the next challenging sentence. If however, the participant chose a poor or partial refutation sentence, the computer provided an exaggerated version of the challenging sentence, thus making it easier for the participant to reject the challenging sentence. The intervention included 10 challenging sentences together with 10 more exaggerated versions, if needed. By giving challenging sentences and preselected refutations for each, there was no need for a face-to-face counselor contact. This is how the system was fully automatized.

#### Procedure and design

**Figure 1.**
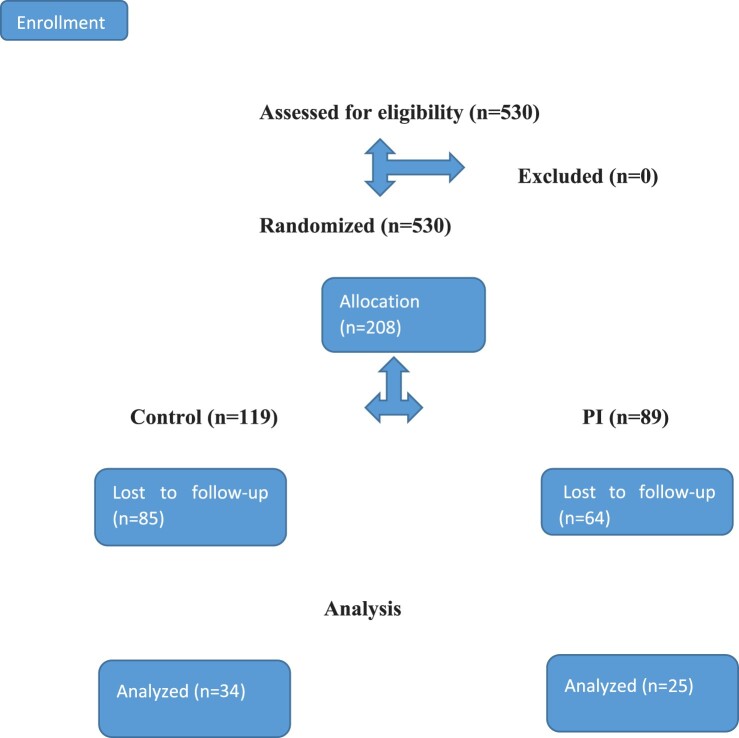
Consort flow diagram- pilot study 1.

Our study included a pilot randomized controlled trial (RCT) design, conducted on computers in a classroom. All outcome measures were administered to the participants at baseline and a month later. Participants were randomized to the control or to the PI condition, using computer-generated random numbers. Both of the groups’ participants had received an electronic link, which directed them to the research. Figure 1 depicts the procedure, following the CONsolidated Standards of Reporting Trials (CONSORT) (http://www.consort-statement.org, 2019).

In phase one, both groups filled in the questionnaire that contained personal background questions. For matching participants’ responses during the two measurement phases of the study, the participants had to provide the last four digits of their national I.D. Providing only the last four digits maintained their anonymity from the research team.

After providing their personal data, the participants had completed the 17 items of the I-CUTE, followed by the Condom use barriers scale. Then, the participants read educational material regarding HIV statistics and prevention methods. At this point, participants were randomized to the control or PI conditions, using an on-line random number generating system (www.randomizer.org). The control group had received 10 questions with True/ False answers, from which they had to choose the correct answer to the best of their knowledge. The experimental group on the other hand, had received the PI intervention, as described above. A month after the first phase, a follow-up assessment was conducted by sending another electronic link to all the participants. In this link, both of the groups had received the same content, which included the I-CUTE and the Condom Use barriers scale.

#### Statistical analysis

We first examined the differences in sociodemographic variables between both group, using t-tests for continuous data, and chi square tests for categorical data. The main analyses were done by paired t-tests on the outcome measures, within each group separately. However since we observed significant group differences on sociodemographic variables (see below), we repeated this test with a mixed design analysis of covariance (ANCOVA). The between-subjects variable was group (control/PI) and the within-subjects variable was time of assessment (pre/post). Age and gender were statistically controlled for. Finally, we calculated each condition’s effect sizes, using Cohen’s d’ index.

### Results

There were no significant differences between both groups on base-line barriers and I-CUTE scores (both *p* > 0.05). However, there were significantly fewer women in the PI group than in the control group (*X*^2^(1) = 9.6, *p* < 0.005). In addition, participants in the PI group were significantly older than the participants in the control group (*t*(57) = 2.39, *p* < 0.05); (see Table 1). Thus, in the ANCOVA described below, the effects of gender and age were statistically controlled for.

**Table 1.** Means and standard deviations (SD) and percentages of main study variables in psychological inoculation (PI) and control groups (study 1).

Group	PI	Control
	*n* = 35	*n* = 24
Variable	Mean (SD)	Mean (SD)
Age	24.04(3.48)	21.82(3.55)*
Barriers T1	66.38(19.80)	65.16(15.06)
Barriers T2	68.85(17.18)	62.68(16.80)
I-CUTE T1	48.20(6.70)	50.73(8.17)
I-CUTE T2	51.72(8.74)*#	51.44(8.15)
*Gender*		
Men	21 (84%)	15(44.1%)
Women	4 (16%)	19(55.9%)*
*Partner’s status*		
No partner	16(64%)	25(73.5%)
One partner w.o. Sex	4(16%)	3(8.8%)
Several partners with Sex	0(0%)	2(5.9%)
One partner with Sex	5(20%)	4(11.8%)

Notes: *p < 0.05; I-CUTE = Indirect Condom Use Test; w.o. = Without.

*#*p* < 0.05 between Pre and Post measures within the PI group.

Table 1 shows the means (SD) of the main study variables per group. Using paired t-tests, within each group separately, results show that the controls did not change significantly over time on barriers (*t*(28) = 0.60, *p* > 0.05) or on I-CUTE scores (*t*(33) = 0.63, *p* > 0.05). In contrast, while participants in the PI group showed no change in barriers (*t*(18) = 0.54, *p* > 0.05), they did show significant increases over time in I-CUTE scores (*t*(24) = 2.62, *p* < 0.05). Table 1 depicts these changes.

We also analyzed these data using an ANOVA, focusing on the Time x Group interaction. In this analysis, there was no significant time × group interaction in relation to I-CUTE scores (*F*(1,57) = 2.61, *p* = 0.11). Though not significant, we examined the effect of time in each group separately, because of the results of the paired t-test. In the control group, time had no effect on I-CUTE scores (*F*(1,33) = 0.39, *p* > 0.05). In contrast, the time had a significant effect on I-CUTE scores in the PI group (*F*(1,24) = 6.84, *p* = 0.01), supporting the paired t-test analysis.

Because groups were significantly different in age and gender, these variables were statistically controlled for, in an additional analysis of covariance (ANCOVA). In this analysis, the expected Time by Group interaction was not significant for barriers (*F*(1,44) = 0.9, *p* > 0.05) and for I-CUTE scores (*F*(1,55) = 0.93, *p* > 0.05). Nevertheless, we examined the effects of time on I-CUTE scores in each group separately, statistically controlling for age and gender. After statistically controlling for age and gender, there was still no time effect in the control group (*F*(1,31) = 0.83, *p* > 0.10). In contrast, in the experimental group, the time effect tended to remain significant after statistically controlling age and gender (*F*(1,22) = 1.82, *p* = 0.07) in relations to I-CUTE scores.

Due to the fact that there was no significant change on the barriers scale in either group, in a post-hoc analysis, we examined the subscales of the barriers scale and analyzed whether there was a significant change over time in one or more of these subscales per group. These subscales were derived from a factor analysis previously conducted (St. Lawrence et al., 1999). These subscales included barriers concerning partners, sexual experience, motivation and access to condoms. No significant changes were found in any of the subscales in either group as well. These results appear in Table 2.

**Table 2.** Changes in barrier subscales per group (study 1).

Group	PI		Control	
	*n* = 35		*n* = 24	
Factor	Mean (SD)	*d*′	Mean (SD)	*d*′
Partner T1	18.47(5.25)		17.46(5.12)	
Partner T2	18.81(4.70)	0.07	17.02(4.95)	0.09
Sexual experience T1	18.57(4.96)		16.73(4.46)	
Sexual experience T2	18.09(5.13)	0.09	17.36(4.16)	0.15
Access T1	19.66(6.10)		20.08(4.99)	
Access T2	20.66(6.09)	0.16	19.23(5.14)	0.17
Motivation T1	11.52(3.51)		10.38(3.25)	
Motivation T2	12.17(4.36)	0.16	10.23(3.57)	0.04

### Discussion

The main findings of Study 1 show that controls showed no change over time on either barriers or condom use tendencies. In contrast, in the PI group, a significant increase in condom use tendencies was found, however, without reductions in barriers.

These results partly support our hypotheses. This fully automatized format of the PI intervention improved condom use tendencies. However, this was not accompanied by a change in cognition, namely the condom use barriers. Furthermore, three important limitations occurred in this study.

The first limitation is that in the current setting, a feedback on participants’ refutation performance was not provided (e.g. ‘you refuted the sentence very well’). This may have reduced the level of learning. The second limitation is that participants of the PI condition received three refutation options, from which they had to choose the strongest refutation sentence. However, providing only three options may have been too simple and this could have reduced the required cognitive efforts. We assume that the combination of both limitations may have prevented a change in barriers in the PI condition.

The third limitation is that the groups were different in the distribution of gender. Study 2 addressed these limitations by adding feedback to participants’ refutation performance, by requiring participants to choose 1 of 5 refutation options rather than choosing only from three (both would increase the depth of learning) and by matching participants on baseline barriers and on gender from each condition. The second reason to match for gender is due to gender differences often seen in psychological interventions in general and with PI specifically (Farchi & Gidron, 2010).

## Study 2

### Method

#### Participants

The study sample was recruited from students at two Belgian Universities and a French University. This study was approved by the ethical committee of one of the universities, which served as the basis for approval in the others. Furthermore, the study was given as part of lectures on behavioral change and were thus linked to students’ study material. All the students were English literate. The minimal age was 18 years old. All students were informed about the study by email and by their program directors, emphasizing their anonymity and voluntary participation. Initially, 147 students entered the website and completed the base-line assessments (Phase one). Of these, 35 completed *both* base-line and the one-month follow-up assessments, which constitutes 23.8% of those initially entering the website. As in Study 1, participants provided their consent to take part in the study electronically.

#### Design and procedure

**Figure 2.**
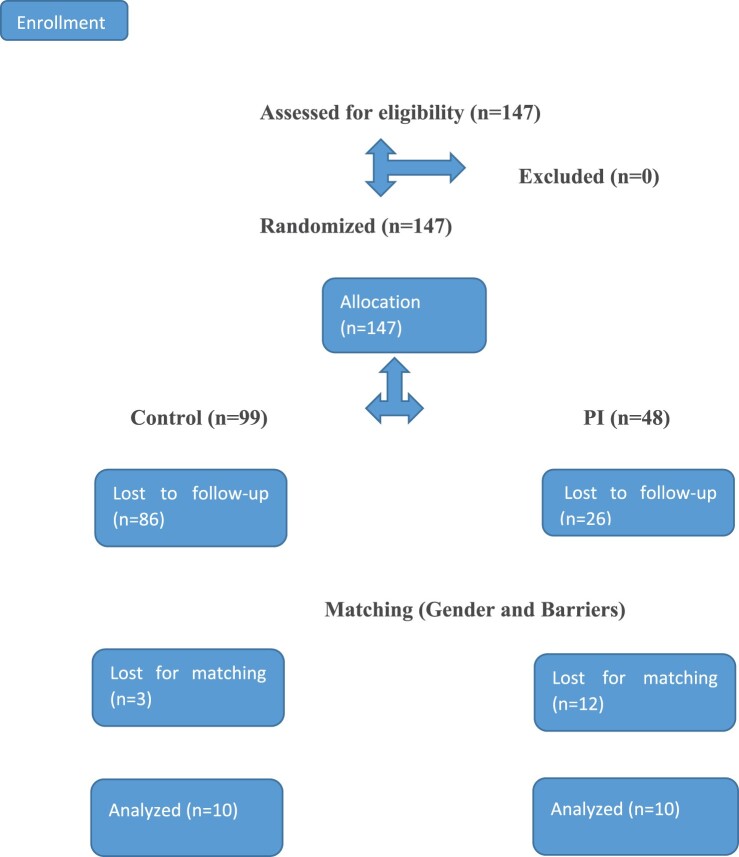
Consort flow diagram- pilot study 2.

In Study 1, we observed high attrition rates in both groups. These could have been partly due to lack of interactivity (Parker, 2011). In Study 2, we increased the interactivity in the PI condition through increasing the cognitive efforts. In contrast, in the control group the interactive element could not be increased. To overcome the expected attrition in controls, we then performed a forced randomization ration of 2:1 in favor of controls (Figure 2).

We initially observed tendencies for group differences in the study outcomes in the full sample (*n* = 35). To clarify this and since the groups also differed significantly on baseline barriers, we decided to match participants on baseline barriers. We additionally matched participants on gender since gender differences have been found in the effects of PI (Farchi & Gidron, 2010). Thus, this second pilot study employed a matched RCT design, which aims to increase statistical power and to ensure group equality on important variables at baseline, after randomization. Of the 35 participants who completed both assessments, we then matched pairs of PI and control participants on gender and baseline barriers, after the study was completed. A similar design by Gidron, Davidson, and Bata (1999) was used. As in Study 1, participants completed baseline and one-month follow-up measures of condom use barriers and condom use tendencies. Figure 2 depicts the procedure following CONSORT. Due to the matching procedure, three controls and 12 PI participants were excluded from the analyses, leaving 10 participants per group.

#### Measures

The measured used in Study 2 were identical to those of Study 1. Background information included age, gender, having a partner, having sexual relations with the partner and using a contraceptive. The outcome measures included the condom use barriers scale (St. Lawrence et al., 1999), and the I-CUTE for assessing condom use tendencies (Levy et al., 2017). In Study 2, the internal reliability of the barriers scale was high (Cronbach’s alpha = .86), as was that of the I-CUTE (Cronbach’s alpha = .84).

#### Interventions

As in Study 1, this study included two groups, an experiment (PI) condition and a control condition (health education). All the components of the PI and the control conditions were identical to Study 1, except for the following.

Unlike Study 1, in Study 2, PI participants received electronic pre-programmed feedback on their refutation performance (e.g. ‘That was a very good refutation’), pending their performance. In addition, the participants had to choose the strongest refutation sentence out of 5 possible refutation sentences, rather than only out of three as in Study 1. These two additional sentences were inserted in between the middle option and the extreme refutation options. This made the identification of the correct refutation sentence more difficult, hence was expected to require greater cognitive effort and thus, to lead to greater reductions in cognitive barriers. Thus, every challenging sentence was followed by 5 possible refutation sentences. The refutation sentences were ordered from the weakest to the strongest refutation. In some statements, the order of the refutation sentences was reversed to increase the cognitive effort and to prevent response sets, as in Study 1. If the participant chose a weak refutation sentence (1–3), the system provided the following feedback in accordance with the strength of the refutation. For instance, if the participant chose the weakest refutation, the system indicated: *Your refutation was very weak!* If the participant chose a medium strength of refutation, the system indicated: *Your refutation was not strong enough!* If the participant chose a sentence reflecting strong refutation (4–5), the system indicated *Your refutation was very strong*! Well done!.

Both groups had received an educational component, which included background information about HIV statistics and prevention. The education content reflected the main element of the control condition. However, controls also received a knowledge test, to control for the interactive aspect of the PI, as in Study 1.

#### Statistical analysis

Due to employing a matched RCT design, we used paired t-tests for testing equality at baseline between groups and for testing changes over time on each outcome, in each group separately. We did not perform an additional mixed design ANOVA because of the matching procedure, where participants from both groups were paired on gender and baseline barriers scores. Thus, paired t-tests seemed more appropriate. Such analyses were performed in other intervention trials (Bartlett et al., 2013; Olley et al., 2011). In all of the following analyses, the effects of the two interventions over time were examined in single tailed tests, because the hypotheses were unidirectional. As in Study 1, we additionally calculated each condition’s effect sizes, using Cohen’s d’ index.

### Ethics statement

The main investigator of this study (Y.G) was working at the time in The Free University of Brussels (VUB). However, since the VUB ethics committee could not approve studies conducted out of Belgium, we obtained permission to conduct this study from the academic director of the participants' program, after submitting a full research proposal.

### Results

We compared the baseline levels (condom use tendencies and barriers) and background data (e.g. age, gender) of participants who initially entered the online system and those who completed the trial. On all study variables, no statistically significant differences were found (all *p*s > 0.05). Thus, the results reported below maybe generalized with caution to all of the potential participants initially entering the online system.

The mean age in both of the groups was 20.8 years old. The SD in the control was 1.99, and in the PI it was 1.75. In both groups, 60% of the participants were females. Thus, no significant differences were found on age and gender between both groups. There were no significant differences between groups in base-line barriers and I-CUTE scores as well (all *p* > 0.05). These validated our matching procedure.

Importantly, I-CUTE levels nearly significantly increased in the PI group (*t*(9) = 1.79, *p* < 0.06) and also tended to do so in controls (*t*(9) = 1.49, *p* < 0.09). In addition, barriers were significantly decreased in the PI group (*t*(9) = 2.01, *p* < 0.05), while in controls, barriers significantly increased over time (*t*(9) = 2.69, *p* < 0.05). Table 3 depicts these changes.

**Table 3.** Means and standard deviations (SD) and percentages of main study variables in psychological inoculation (PI) and control groups (study 2).

Group	PI		Control	
	*n* = 10		*n* = 10	
Variable	Mean (SD)	*d*′	Mean (SD)	*d*′
IC-Pre	52.40(6.31)		50.90(6.72)	
IC-Po	56.00(7.54)	0.52	53.80(5.78)	0.46
Barr-Pre	61.90(8.93)		60.40(9.24)	
Barr-Po	56.40(7.87)	0.65	66.10(9.11)	0.62

Notes: IC = Indirect Condom Use Test; Pre = pre test; Po = Post test C = control; Bar = Barriers; PI = Psychological Inoculation.

As in Study 1, we then explored the changes in the barriers sub-scales based on St. Lawrence et al. (1999). We found significant changes in two of the sub-scales, namely ‘Partner’ and ‘Effects on Sexual experience’. The sub-scale of ‘Partner’ refers to the relationships of the participant with his/her partner as it comes to issues of trust or intimacy. The sub-scale of ‘Effects on Sexual experience’ refers to the feelings and thoughts one may have about the condom itself. Only in the PI group, significant reductions were seen on the ‘Partner’ sub-scale (*t*(9) = 2.77, *p* < 0.05) and on the ‘Effects on Sexual experience’ sub-scale (*t*(9) = 2.53, *p* < 0.05). No other changes in barriers sub-scales were found in the PI group, and no changes were found on any of the sub-scales in the controls (all *p* > 0.05). Table 4 depicts these changes. Finally, degree of refutation in the PI group was significantly and positively correlated with change in I-CUTE scores (*r* = 0.55, *p* = 0.05).

**Table 4.** Changes in barrier subscales per group (study 2).

Group	PI		Control	
	*n* = 10		*n* = 10	
Sub-scale	Mean (SD)	*d′*	Mean (SD)	*d′*
Partner T1	18.60(2.45)		17.00(3.77)	
Partner T2	15.80(2.57)*	1.11	18.60(3.81)	0.42
Sexual experience T1	15.70(2.62)		16.10(3.60)	
Sexual experience T2	13.90(2.37)*	0.72	16.50(4.00)	0.10
Access T1	18.40(4.29)		18.40(2.37)	
Access T2	17.00(3.16)	0.37	19.10(3.92)	0.22
Motivation T1	9.20(2.48)		8.90(2.72)	
Motivation T2	9.70(2.41)	0.20	10.50(2.79)	0.58

*p < 0.05.

### Discussion

The main results of Study 2, show a tendency for improvement of I-CUTE scores in the PI condition and a weaker tendency in controls. In addition, while in the PI condition, a significant decrease in condom-use barriers was found, in the controls barriers significantly worsened over time. Exploring the barrier sub-scales revealed that a significant reduction both in the ‘Partner’ and in the ‘Effects on Sexual experience’ sub-scales was observed only in the experimental PI condition.

Importantly, the degree of refutation of the challenging sentences in the PI group was significantly and positively correlated with change in I-CUTE scores. These findings replicate those of Dorling, Blervacq, and Gidron (2018), found in relation to physical activity, and support the importance of refuting challenging sentences to create behavioral change later. The findings of studies 1 and 2 will now be discussed in depth.

## General discussion

The aims of these two pilot RCT studies were to test the feasibility and preliminary effects of a fully automatized PI intervention on reducing barriers for condom use and on increasing tendencies to use condoms. The results of Study 1 showed a significant increase in condom use tendencies as measured by the valid I-CUTE tool, only in the PI group, supporting our hypothesis. However, no change was found in the condom use barriers scale in either group. The results of Study 2 showed a significant increase in condom-use barriers but a tendency for increased condom use tendencies among the controls. In contrast, in the PI condition of Study 2, a significant reduction in barriers was observed, as well as a strong tendency to increase condom use tendencies. Importantly, among the PI participants, a significant and positive correlation was observed between the strength of refuting the challenging sentences, the main essence of the PI, and increases in condom use tendencies

Each of the results of both studies, partly support our hypotheses. However, questions need to be raised concerning the differences between the two studies. One main difference is the lack of effects of PI on barriers in Study 1, while these effects were observed in Study 2. Specifically, in Study 2 we found a significant reduction in two of the barrier sub-scales, namely in the ‘Partner’ and ‘Effects on Sexual experience’ barriers, only in the PI group. These differences may result from modifications in the automatized program which were performed in Study 2. These included adding two more refutation sentences on top of the three given in Study 1, possibly resulting in deeper cognitive efforts, on behalf of the participant. Furthermore, participants in Study 2 were provided with feedback on their refutation performance. Both modifications might have increased learning, manifested by reduced barriers only in Study 2, in the PI condition.

The reduction in barriers seen in Study 2 replicate the findings of Olley et al. (2011) regarding condom use barriers and extend them to non-Sub Saharans in Study 2. The fact that the strength of refutation was correlated with behavioral tendencies to use condoms (Study 2), replicates the findings of Dorling et al. (2018). The later study found that the strength of refuting challenging sentences predicted the degree of performing physical activity two months later. This is a major aspect of social cognitive models in general and of the PI method specifically, where by modifying one’s cognitive biases, health behavior will then change for the better.

The findings showing which barriers sub-scales were reduced (‘Partner’ and ‘Effects on Sexual experience’) and not other sub-scales (‘Availability’ and ‘Motivational barriers’), need to be discussed. We speculate that the challenging sentences of the PI condition may have targeted to a greater extent the two sub-scales which were modified. Furthermore, we speculate that the unchanged ‘Availability’ sub-scale reflects more objective and situational factors (Price, religion etc.), which are more difficult to modify and do not only reflect one’s perceptions and cognitive biases.

Surprisingly, in Study 2, controls evidenced a worsening of barriers compared to improvements seen in the PI condition. We speculate that the mere exposure of controls to the measure of barriers twice, without addressing them (unlike in the PI), may have ‘primed’ them and increased their awareness, and thus made them appraise the barriers as more severe. In contrast, PI participants were systematically guided to refute such barriers, and this was manifested by the reduction in barriers despite the repeated exposure.

Unexpectedly, both groups in Study 2 showed a tendency towards increases in condom use scores. This was not seen in Study 1, were condom use scores increased only in the PI group. It might be related to methodological differences in sample size and the use of matching in Study 2. Yet, neither PI nor control participants showed significant increases in condom use. However, the fact that the level of refuting the challenging sentences predicted condom-use tendencies in the PI condition suggests that such increases in that group occurred because of the PI intervention. Nevertheless, corroboration of our findings by future additional data with larger samples would lend support and clarify these findings.

It is difficult to distinguish whether the different findings of the two studies are a result of the improved methodology of Study 2, or due to the different cultural settings. While we did not change the challenging sentences between the studies, in order to keep the generalizability of testing the interventions’ feasibility, future studies may consider adapting the challenging sentences to the barriers of each culture, in order to increase the effectiveness of the PI.

### Limitations

The present studies included a few limitations. Accessibility to computers and the internet is not widespread in the targeted (Sub-Saharan) population of the sample described in Study 1. However, the present study was ‘a proof of concept’, where we aimed to preliminarily test the fully computerized PI intervention, with the understanding that if this has positive effects, it could be applied on a larger scale, to people with computer or internet access. In addition, such a program could also be mounted on mobile phones, which are widely used in developing countries.

Moreover, past studies show that self-efficacy, an important component within the Theory of Planned Behavior, may affect intention to perform behavioral changes (Williams, Kessler, & Williams, 2015). However, self- efficacy was not assessed in the studies presented here, an outcome which was found to be increased by the PI in the previous study mentioned above (Olley et al., 2011).

Another limitation is the absence of comparing the computerized automatized method to ‘traditional’ face-to-face PI interventions with a counselor. While this needs to be done in future studies, the aim of the current study was to test the PI versus its usual control, namely education, when both are provided in an automatized format without a counselor. The results of both studies reveal that this is feasible.

Additionally, while performing the trial through computers increased anonymity and reduced social pressure on one hand, this reduced the ability to monitor the social context in which participants did the study, on the other hand (e.g. alone or at home).

Both studies evidenced high rates of drop-out in the follow-ups. However, we did not find any differences between completers and non-completers in study 2, suggesting that we can generalize with caution the findings of that study to all participants initially entering the computerized system. Nevertheless, the high rates of attrition could have resulted from several reasons. It could result from students having other priorities, lack of interest, technical problems or due the sensitive issue, which was investigated, among other reasons. The high rate of attrition and the fact that this study was a preliminary trial, also limited our ability to conduct a longer follow-up.

In addition, we did not conduct an intention to treat (ITT) analysis. Such an analysis may have masked any potential effects in exploratory pilot clinical trials, and was thus, not performed.

In Study 1, gender was not equally distributed between groups. For this reason and due to the importance of gender in this topic and due to gender differences in the effects of PI (Farchi & Gidron, 2010), we matched participants also on gender in Study 2. However, due to the small number of men and women, we could not perform the analysis for each gender separately. Indeed, one past study found better effects of PI in men than in women, in another context (Farchi & Gidron, 2010.) Gender as described above is a substantial element in the Sub-Saharan context. Thus, future studies should conduct stratification before randomization and evaluate the effects of PI versus health education in each gender separately.

A final limitation is the fact that this form of intervention used IT, and required high literacy, which is unsuitable to many rural communities. Nevertheless, a major advantage of the PI, is its versatile flexibility to adapt its contents (challenging sentences) to each culture’s specific barriers and social pressures concerning a health behavior**.** Due to the wide spread use of mobile phones in rural world regions, future studies should test the effects of PI on health behaviors, given via such technology.

Despite these limitations, the present feasibility studies has several strong methodological points and conceptual strengths. First, it used randomized controlled trial designs, which reduce multiple biases. Second, Study 2 used a matched RCT design, which further increases the statistical power. Third, we used a novel indirect and valid measure of condom use tendencies, which bypasses problems of social desirability (Levy et al., 2017). This issue is crucial in such socially sensitive topics. Furthermore, we were able to test this new approach in two different cultural settings. This shows that the online automatized method might be suitable for multiple cultures, though adapting its content to culture-specific barriers may be needed. Finally, because our method is fully automatized and not requiring a counselor/therapist, it can potentially reach large populations if our results will be replicated. This is crucial for a global pandemic such as HIV and for multiple other acute and chronic diseases (e.g. heart diseases, obesity, cancer), in which behavior is a crucial risk factor. Should the results observed in the present two feasibility studies be replicated in larger samples, this novel form of automatized PI may have important implications for HIV prevention on larger scales in the future.

## Data Availability

The data that support the findings of this study are available on request from the corresponding author, [E.L]. The data are not publicly available due to [restrictions e.g. their containing information that could compromise the privacy of research participants].

## Abbreviations

**Abbreviations:** ART: anti-retroviral therapy; HBM: health belief model; HIV: human immunodeficiency virus; PI: psychological inoculation; PLHIV: people living with HIV; PrEP: pre-exposure prophylaxis; SCM: social cognitive model; SSA: sub-Saharan Africa; TPB: theory of planned behavior
